# *EHD4* and *ASAP2* are critical negative regulators of the claudin‐5‐based endothelial barrier

**DOI:** 10.1111/febs.70357

**Published:** 2025-12-08

**Authors:** Yosuke Hashimoto, Gergő Porkoláb, Natalie Hudson, Jeffrey O'Callaghan, Nicole Hanley, Conor Delaney, Mária A. Deli, Peter Westenskow, Matthew Campbell

**Affiliations:** ^1^ Smurfit Institute of Genetics Trinity College Dublin Dublin 2 Ireland; ^2^ Graduate School of Biomedical and Health Sciences Hiroshima University Hiroshima Japan; ^3^ Institute of Biophysics, Biological Research Centre Eötvös Loránd Research Network Szeged Hungary; ^4^ Roche Pharma Research and Early Development, Roche Innovation Center F. Hoffmann‐La Roche AG Basel Switzerland; ^5^ FutureNeuro, Science Foundation Ireland Research Centre for Chronic and Rare Neurological Diseases, Royal College of Surgeons in Ireland University of Medicine and Health Sciences Dublin Ireland

**Keywords:** angiogenesis, ASAP2, Claudin‐5, EHD4, endothelial barrier, tight junctions

## Abstract

Disruption to barriers of the central nervous system (CNS) has been shown in both prime and drive pathologies observed across numerous neurological and ophthalmological conditions. These barriers are composed of well evolved endothelial tight junctions, and the key junctional component, claudin‐5 (CLDN‐5), is responsible for maintaining homeostasis of brain and retinal tissues. Indeed, decreased CLDN‐5 expression has now been observed across many neurological and retinal diseases. Additionally, methods aimed at stabilising and upregulating CLDN‐5 expression may have profound efficacy in treating a vast array of these conditions. However, very few targeted and specific methods can enhance CLDN‐5 expression levels, and none of these have detailed its localisation and stability on the cell surface. In an effort to discover unknown and specific regulators of CLDN‐5 expression, we performed a genome‐wide cell‐sorting‐based phenotypic screen using CRISPR/Cas9. Sorting cells based on the phenotype of ‘barrier tightness’ revealed two candidate genes, EH domain‐containing protein 4 (*EHD4*) and Arf‐GAP with SH3 domain, ANK repeat, and PH domain‐containing protein 2 (*ASAP2*), which, when suppressed, led to significant upregulation of CLDN‐5 protein on the cell surface. *EHD4* appeared to regulate the transcriptional activity of *CLDN5*, whereas *ASAP2* controlled junctional localisation of CLDN‐5. Identification of these candidate genes suggests that pharmacological inhibitors of *EHD4* or *ASAP2* may represent profound approaches to regulating CLDN‐5 in neural endothelial cells.

## Graphical abstract

Cell‐surface CLDN‐5 protein levels can be evaluated using a probe that can bind to the extracellular domains of CLDN‐5. A probe derived from *Clostridium perfringens* enterotoxin allows us to isolate cells with high CLDN‐5 protein levels from a knockout cell library. We discovered two previously unknown negative regulators of the CLDN‐5 protein using this system: *EHD4* and *ASAP2*. The inhibition of these genes could stabilise CLDN‐5‐based endothelial barriers.

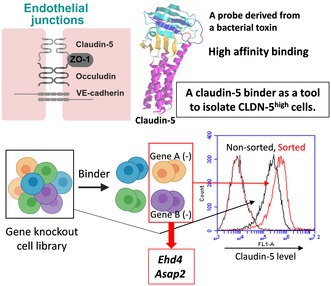

**Abbreviations**
AMPK: AMP‐activated protein kinase
*ASAP2*
: ArfGAP with SH3 domain, ankyrin repeat and PH domain 2
BBB: blood–brain barrier
C‐CPEmt: A mutant of C‐terminal fragment of *Clostridium perfringens* enterotoxin
CLDN‐5: claudin‐5
CNS: central nervous system
CRISPR: clustered regularly interspaced short palindromic repeats
DMEM: Dulbecco's modified Eagle's medium
ECs: endothelial cells
EHD: EH domain‐containing
EndMT: endothelial‐to‐mesenchymal transition
FBS: fetal bovine serum
FD: fluorescein isothiocyanate‐dextran
FPKM: fragments per kilobase of transcript per million mapped reads
GLUT1: glucose transporter‐1
gRNA: guide RNA
GSK‐3β: glycogen synthase kinase‐3β
HUVEC: human umbilical vein endothelial cells
iBRB: inner blood‐retina barrier (iBRB)
IGF: insulin‐like growth factor
JAM: junctional adhesion molecule
KO: knockout
MFI: mean fluorescence intensity
OE: overexpression
PI3K: phosphatidylinositol 3‐kinase
S1P: sphingosine‐1‐phosphate
TEER: trans‐endothelial electrical resistance
TEM: tumoUr endothelial marker
TGF‐β: transforming growth factor‐β
TJs: tight junctions
TLL1: Tolloid‐like 1
T‐TBS: tris‐buffered saline containing 0.05% Tween‐20
VE‐: vascular endothelial (VE)‐
VEGF: vascular endothelial growth factor
ZO‐1: zonula occludens‐1

## Introduction

The neural tissues of the retina and brain require a constant supply of nutrients and oxygen while maintaining highly restrictive barriers against potentially harmful systemically derived molecules. In order to maintain such selectivity, brain and retinal microvascular endothelial cells (ECs) have very well‐developed tight junctions (TJs) and low rates of fluid phase transcytosis. TJs are located along the entire inter‐endothelial cleft and limit the passive paracellular diffusion of ions, solutes and macromolecules. Collectively, the properties associated with these ECs are termed the blood–brain barrier (BBB) and in the retina, they are referred to as the inner blood‐retina barrier (iBRB).

Claudins (CLDN) are tetra‐transmembrane proteins that build TJs by oligomerisation of their extracellular domains. The predominant CLDN in TJs of the BBB and iBRB is CLDN‐5 [1]; CLDN‐5 is categorised as a barrier‐forming CLDN that makes strong TJs with low ion and solute permeabilities. Most CLDNs, including CLDN‐5, have a binding motif to ZO‐1 (zonula occludens‐1), which is the major scaffold protein for TJs, allowing for stabilisation of their junctional localisation by connecting to actin cytoskeleton [2]. Other endothelial junctional components—such as connexin‐43 in gap junctions and vascular endothelial (VE)‐cadherin in adherens junctions—stabilise inter‐endothelial junctions via binding to ZO‐1 and other scaffold proteins [3, 4].

A study using CLDN‐5 knockout mice has revealed that CLDN‐5 is responsible for regulating size‐selectivity at the BBB to maintain neural homeostasis [5]. Numerous studies have now demonstrated that decreased CLDN‐5 expression and increased vascular permeability can initiate or precede a large range of neurological and retinal conditions including cognitive decline, depression, psychosis, seizures and retinal neovascularisation [6, 7, 8, 9, 10, 11]. Added to this, missense mutations in *CLDN5* were recently shown to cause epilepsy, brain calcification, and microcephaly [12, 13]. It is known that traditional mood‐stabilisers such as lithium and valproic acid can enhance CLDN‐5 expression by partial inhibition of the glycogen synthase kinase‐3β (GSK‐3β) pathway that is one of the well‐known CLDN‐5 regulatory pathways [14, 15]. Additionally, the small molecule, RepSox, which is a potent transforming growth factor‐β (TGF‐β) signalling inhibitor, has been shown to stabilise the BBB in mice and attenuate the susceptibility to kainic‐acid‐induced seizures [6]. That said, upregulating CLDN‐5 levels and driving microvascular stabilisation is now considered a powerful therapeutic strategy for a large range of conditions [6, 8, 11].

Reporter cell‐based approaches have traditionally been very powerful tools to identify novel chemical and/or genetic regulators of target transcripts. However, for membrane bound proteins, sometimes the identified regulators do not always predict the final cell‐surface expression/localisation levels of the target proteins. In this regard, cell sorting‐based phenotypic screening approaches can be considered more useful to identify the key genes that regulate the transcription, posttranslational modification and ultimate subcellular localisation of membrane proteins. In order to conduct these screens, it is essential to first have a molecular probe that can label cells without fixation and permeation [16, 17, 18]. In effect, establishing the phenotype of ‘barrier tightness’ or ‘barrier weakness’ can then allow one to examine the transcriptional regulators of such a phenotype.

In the case of CLDN‐5, the cell‐surface expression of the protein is not only controlled by transcriptional regulators, but also by the junctional localisation/stability and the actin cytoskeleton. A modified C‐terminal fragment of *Clostridium perfringens* enterotoxin (C‐CPEmt) has previously been shown to bind to the extracellular domain of CLDN‐5 with high affinity [19, 20]. Therefore, we hypothesised that this probe, C‐CPEmt, could be used as a molecular tool to detect cells expressing high levels of CLDN‐5 (CLDN‐5^high^ cells) from a knockout cell library, and that this genome‐wide screen could be used to discover novel regulatory genes for CLDN‐5 and thereby ‘barrier tightness’. Here, we describe the identification of two novel negative CLDN‐5 regulators, *EHD4* and *ASAP2*. We show that *EHD4* functions as an upstream transcriptional regulator and *ASAP2* functions as a junctional stabiliser. These targets represent potent therapeutic targets for ophthalmological and neurological diseases where barrier stabilisation and regulation will be disease‐modifying.

## Results

### FACS‐based phenotypic screen for detecting regulatory genes driving CLDN‐5 expression

**Fig. 1:**
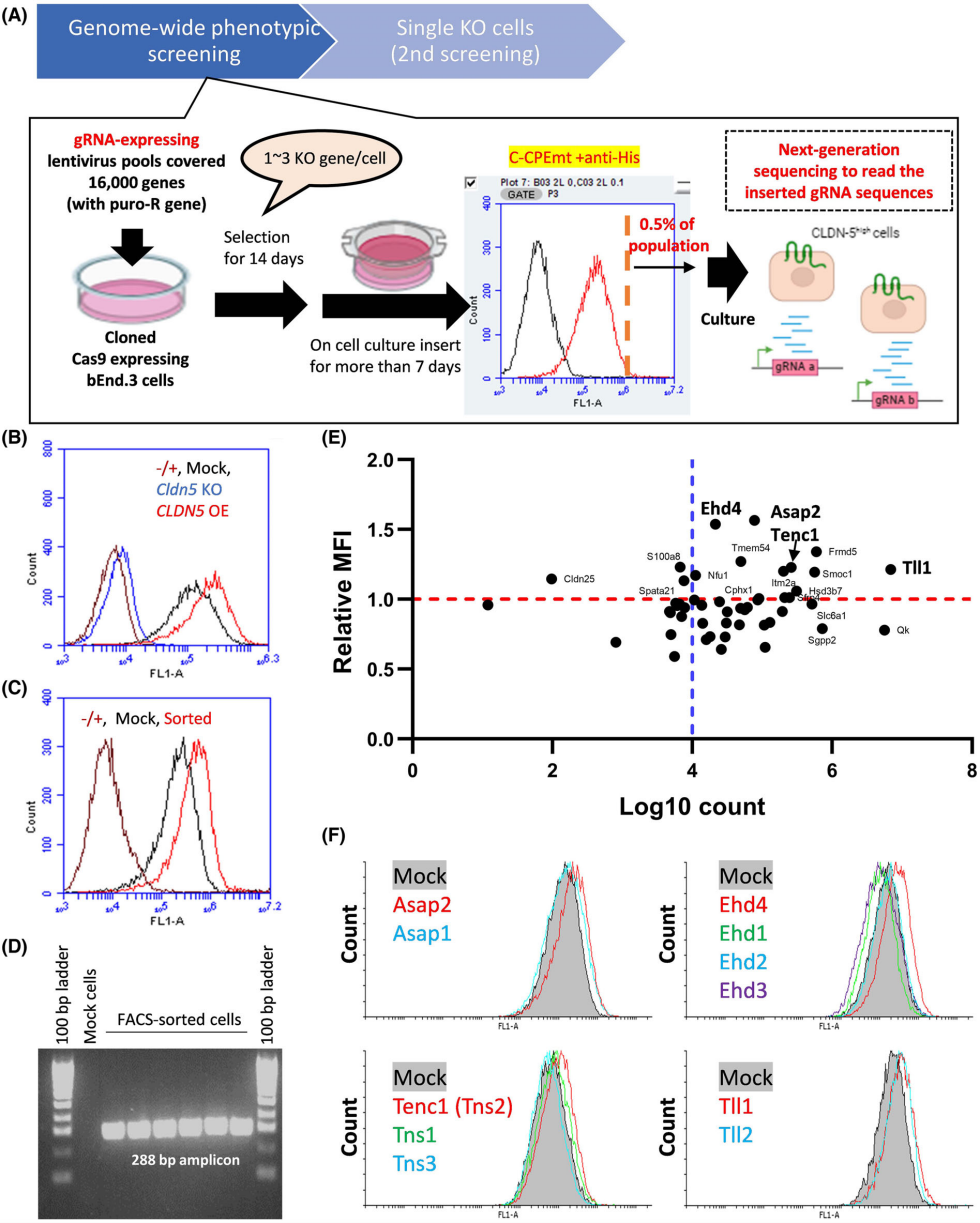
CRISPR/Cas9‐mediated phenotypic screen to identify CLDN‐5/CLDN‐5 regulatory genes. (A) Schematic illustration of the genome‐wide phenotypic screen using CRISPR/Cas9. bEnd.3/Cas9 cells were transduced with a lentiviral guide RNA (gRNA) library and non‐infected cells were removed by puromycin. After selection, cells were maintained on cell culture inserts for seven days to maximise the cell‐surface CLDN‐5 expression. Then, cell‐surface CLDN‐5 was labeled by 10XHis‐tagged C‐CPEmt (a mutant of C‐terminal fragment of *Clostridium perfringens* enterotoxin) and fluorescently labeled secondary antibodies and a population of 1% of the total cells with the highest fluorescence intensity was collected by FACS. Genomic DNAs were harvested from sorted cells, and inserted gRNAs in genome DNAs were analysed by next‐generation sequencing. Detected gRNA sequences were used to prepare single gene knockout (KO) bEnd.3 cells and their cell‐surface CLDN‐5 expression was analysed as a secondary screening. (B) bEnd.3 cells expressing gRNA against mouse CLDN‐5 (CLDN‐5 KO cells) or overexpressing (OE) human CLDN‐5 (CLDN‐5 OE cells) were used to analyse a CLDN‐5 dependency for C‐CPEmt‐binding to bEnd.3 cells (*n* = 1). (C) Cell‐surface CLDN‐5 expression levels after one round of sorting. Cells were maintained on cell culture inserts for seven days before flow cytometric analysis was carried out using 10XHis‐tagged C‐CPEmt (*n* = 1). (D) PCR amplification for gene cassettes derived from gRNA‐expressing lentivirus. PCR products from genome DNAs of non‐infected cells (mock) and sorted cells were loaded to ethidium bromide‐containing 2% agarose gel. (E) Scatter plot of total counts of gRNAs detected by next‐generation sequencing in three independent screens versus mean fluorescence intensity (MFI) of single gene KO bEnd.3 cells stained by 10XHis‐tagged C‐CPEmt (*n* = 1). The MFI of the KO cells is normalised to the MFI of mock cells to express the relative cell‐surface CLDN‐5 expression level. Red dash line indicates the relative MFI is 1 and blue dash line indicates a cut‐off value for the count detected by next‐generation sequencing. (F) Cell‐surface CLDN‐5 expression levels of single gene KO bEnd.3 cells. Plasmids expressing gRNAs against candidate genes and their major family members were transfected into bEnd.3/Cas9 cells. Cells were maintained in cell culture flasks for seven days at confluent states before flow cytometric analysis was carried out using 10XHis‐tagged C‐CPEmt (*n* = 1).

To identify novel CLDN‐5 regulators, we sought a different screening method from traditional reporter‐based screens, such as a screen performed by Roudnicky et al. [21], and decided to conduct a phenotypic screen using a pooled CRISPR library (Fig. 1A). Here, we used an immortalised mouse–brain EC line, bEnd.3 cells to make single colony‐derived CRISPR/Cas9‐expressing cells (bEnd.3/Cas9). As a binding probe, we prepared C‐CPEmt that could bind to many CLDN family members including CLDN‐5, but its binding to bEnd.3 cells was predominantly CLDN‐5‐dependent as CLDN‐5 is the predominant CLDN in bEnd.3 cells and C‐CPEmt could not bind to bEnd.3/CLDN‐5 KO cells, which are bEnd.3/Cas9 cells expressing guide RNA (gRNA) against CLDN‐5 (Fig. 1B).

Subsequently, a knockout pool of bEnd.3 cells was prepared by infecting a lentiviral library of single gRNAs targeting more than 16 000 genes with six distinct gRNA sequences and 1000 non‐targeting control gRNAs. To mature the intercellular junctional complexes and ensure the cell‐surface expression of CLDN‐5, a knockout pool of bEnd.3 cells were cultured on cell‐culture inserts for seven days. Cells were then harvested and treated with C‐CPEmt to isolate CLDN‐5^high^ cells by FACS.

The isolated cells were then seeded on cell‐culture inserts again and cell‐surface CLDN‐5 expression was evaluated. A one‐round of cell sorting was enough to enrich CLDN‐5^high^ cells (Fig. 1C) as a pronounced shift in the histogram of sorted cells was observed. Genomic DNA was isolated to examine which gRNA sequences were inserted into the genome by reading the sequences around the gRNAs (Fig. 1D), using next‐generation sequencing. This screening was performed three times. In total, gRNAs against 48 genes were detected, but only nine genes (*ASAP2*, *CDC37l1*, *EHD4*, *FLRT1*, *FRMD5*, *HSD3B7*, *KAT6B*, *SFRP4*, *TLL1*) were detected in all three independent runs (Table S1) with no negative controls detected (Fig. 1E). To confirm their knockout‐mediated effect, stably transfected single gene knockout (KO) cells were prepared by transfecting gRNA‐expressing vectors into bEnd.3/Cas9 cells, and cell‐surface expression levels of CLDN‐5 were subsequently evaluated (Table S1). A 1.2‐fold increase in the mean fluorescent intensity as compared to mock gRNA transfected bEnd.3/Cas9 cells was considered a significant difference. Knockout of eight genes (*ASAP2*, *EHD4*, *FRMD5*, *TMEM54*, *S100A8*, *TENC1*, *TLL1*, *ITM2A*) showed a significant upregulation of cell‐surface CLDN‐5 levels (>1.2‐fold changes), while knockout of 30 identified genes did not induce obvious CLDN‐5 upregulation (<1.2‐fold changes) with another 10 identified genes attenuating cell‐surface CLDN‐5 expression level (Table S1). Unfortunately, levels of mRNA for the identified genes *FRMD5*, *TMEM54, ITM2A* AND *S100A8* could not be detected by conventional quantitative PCR (data not shown) and these genes were subsequently excluded from further analysis. All KO cells did not show apparent differences in cell body size or growth speed except for *SDK1* KO cells which had clearly smaller cell bodies (data not shown). To confirm the selectivity of *ASAP2*, *EHD4*, *TENC1* and *TLL1*, gRNAs against their major family members were also transfected into bEnd.3/Cas9 cells and cell‐surface CLDN‐5 expression levels were compared. Except for *TLL1*—upon knockout—the other major family members did not significantly upregulate CLDN‐5 expression levels (Fig. 1F).

### Barrier properties of cells with knockout of candidate genes

**Fig. 2:**
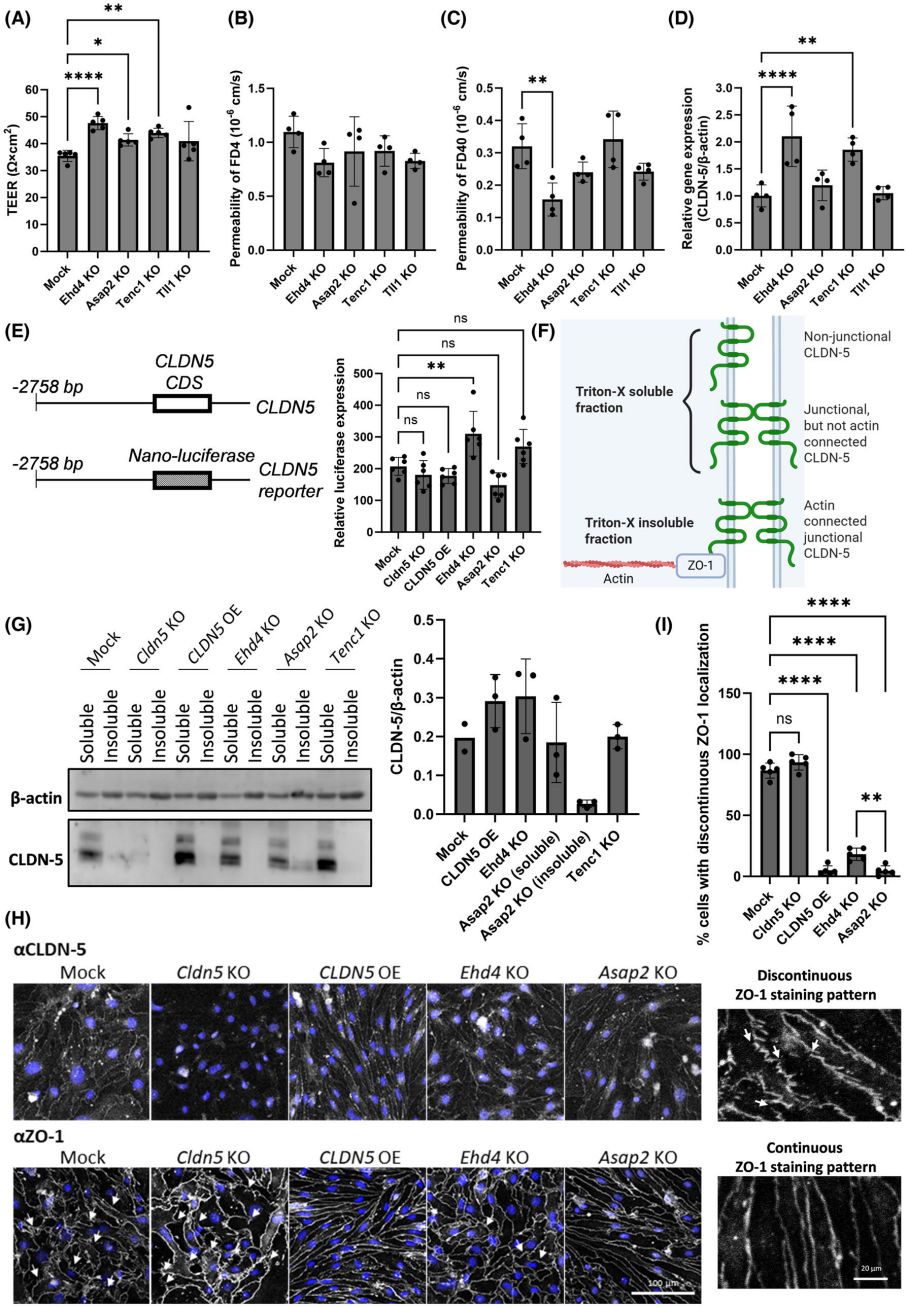
Barrier phenotypes of bEnd.3 cells with knockout of candidate genes. Non‐targeting gRNAs (guide RNAs) (mock) or gRNAs targeting gRNAs‐expressing bEnd.3 cells were cultured for more than seven days to prepare a cell monolayer. (A) TEER (trans‐endothelial electrical resistance) and the permeability of (B) 4 kDa or (C) 40 kDa of fluorescence‐conjugated dextran (FD4 or FD40) across the monolayers and (D) relative CLDN‐5 mRNA levels were measured. Values represent the mean ± SD (*n* = 5 or 4). One‐way ANOVA followed by Dunnett's multiple comparisons test: *P < 0.05; **P < 0.005; ****P < 0.0001. (E) Dual‐luciferase assay to check *CLDN5* transcriptional activity in gene knockout cells. Vector information for plasmid encoding CLDN‐5 upstream gene with coding sequence of nano‐luciferase is shown in left panel, and nano‐luciferase expression level normalised by fire‐luciferase level is shown in right bar graph. Values represent the mean ± SD (*n* = 6). One‐way ANOVA followed by Dunnett's multiple comparisons test: **P < 0.005. (F) Schematic illustration to explain the CLDN‐5 status in 1% Triton‐X‐soluble fractions or ‐insoluble fractions of cells. (G) CLDN‐5 abundance in 1% Triton‐X‐soluble fractions or ‐insoluble fractions of cells. A representative blot is shown in the left panel (three independent experiments were performed) and relative abundance of CLDN‐5 compared to β‐actin in each fraction determined by densitometric image analysis is shown in right bar graph. Values represent the mean ± SD (*n* = 2 or 3). (H) Immunocytochemistry of CLDN‐5 and ZO‐1. The cells were counterstained with Hoechst33342. Discontinuous ZO‐1 is indicated by arrows and enlarged images are shown. At the arrows, ZO‐1 staining is a discontinuous ‘zig‐zag’ pattern. The scale bar indicates 100 μm in representative images and 20 μm in enlarged images. (I) The % of cells with discontinuous ZO‐1 staining at cell–cell border was analysed. Values represent the mean ± SD (*n* = 5). One‐way ANOVA followed by Tukey's multiple comparisons test: **P < 0.005; ****P < 0.0001. CDS, coding DNA sequence; KO, knockout; OE, overexpression.

To validate whether inhibition of the identified candidate genes could enhance the barrier properties of bEnd.3 cells, KO cells were cultured and trans‐endothelial electrical resistance (TEER), which is a representative barrier indicator, and paracellular permeability of tracer molecules (4 and 40 kDa) were measured. Except for *TLL1* KO cells, TEER levels were significantly increased compared to cells expressing negative control gRNAs (mock) (Fig. 2A). These KO cells could not tighten the barrier against molecules < 4 kDa (Fig. 2B), but *EHD4* KO cells significantly tightened the barrier against molecules larger than 40 kDa (Fig. 2C). CLDN‐5 mRNA levels were also significantly upregulated in the monolayer of *EHD4* KO and *TENC1* KO cells, but not in the others (Fig. 2D). These results indicate that inhibition of *EHD4* and *TENC1* may upregulate cell‐surface CLDN‐5 with upregulation of CLDN‐5 mRNA, while inhibition of *ASAP2* and *TLL1* may induce an upregulation of cell‐surface CLDN‐5 with increased CLDN‐5 protein stability/localisation. To verify whether the inhibition of *EHD4* and *TENC1* upregulated the transcriptional activity of *CLDN5*, a promoter‐based dual‐luciferase assay was performed using a nano‐luciferase reporter vector carrying the human CLDN‐5 promoter (Fig. 2E). After transfection of the reporter plasmid into the series of bEnd.3 cells, only *EHD4* KO cells showed significantly higher luciferase activity, indicating that *EHD4* likely controls the activity of upstream transcriptional factors for the CLDN‐5 promoter.

To address the possibility that knockout of these genes changed the membrane microdomains of CLDN‐5 to enhance junctional stability, the abundance of CLDN‐5 in 1% Triton‐X‐soluble and ‑insoluble fractions of cells was examined. CLDNs can form TJs without connecting to the actin cytoskeleton via ZO‐1, but these TJs are considered to be less stable [22]. At this concentration of Triton‐X, CLDN‐5 in the insoluble fraction is considered to be strongly associated with ZO‐1 (a junctional scaffold protein) and actin cytoskeleton (Fig. 2F). In this regard, CLDN‐5 in cells was mainly extracted in the Triton‐X‐soluble fraction and CLDN‐5 abundance in *EHD4* KO cells was higher than that in *ASAP2* KO or *TENC1* KO cells (Fig. 2G). However, it must be noted that *CLDN‑5* in *ASAP2* KO cells was also enriched in the Triton‐X‐insoluble fraction. Of note, CLDN‐5 in bEnd.3 cells overexpressing human CLDN‐5 (CLDN‐5 OE cells) was not enriched in the Triton‐X‐insoluble fraction, indicating that different molecular pathways are required to stabilise junctional CLDN‐5. These results clearly indicate that cell‐surface expression of CLDN‐5 is increased by transcriptional inhibition of *EHD4*, while the connection of CLDN‐5 into actin cytoskeleton to form stronger TJs is promoted by knockout of *ASAP2*.

To examine the junctional stability of these cells, immunolocalisation of CLDN‐5 and ZO‐1 was evaluated (Fig. 2H). Except for CLDN‐5 KO cells, CLDN‐5 was mainly localised at the cell–cell border in these cells. The staining pattens of ZO‐1 were continuous and linear in almost all *CLDN5* OE and *ASAP2* KO cells, but were a ‘zig‐zag’ line with several break points (indicated by arrows in Fig. 2H) at their cell–cell border of almost all mock cells and CLDN‐5 KO cells and some *EHD4* KO cells (Fig. 2I). CLDN‐5 could not be connected to actin cytoskeleton at the points where ZO‐1 scaffold is missing or unstable. Since the presence of CLDN‐5 also stabilises junctional ZO‐1 localisation, ZO‐1 was cytosolically distributed in mock cells and CLDN‐5 KO cells, while it was mainly localised in the cell–cell borders in the other cells. The junctional ZO‐1 was stabilised in *ASAP2* KO cells without the upregulation of CLDN‐5 mRNA, indicating that *ASAP2* inhibition may promote CLDN‐5 stability by connecting to ZO‐1/actin cytoskeleton in bEnd.3 cells.

### Mechanism of action of *EHD4* and *ASAP2* inhibition

**Fig. 3:**
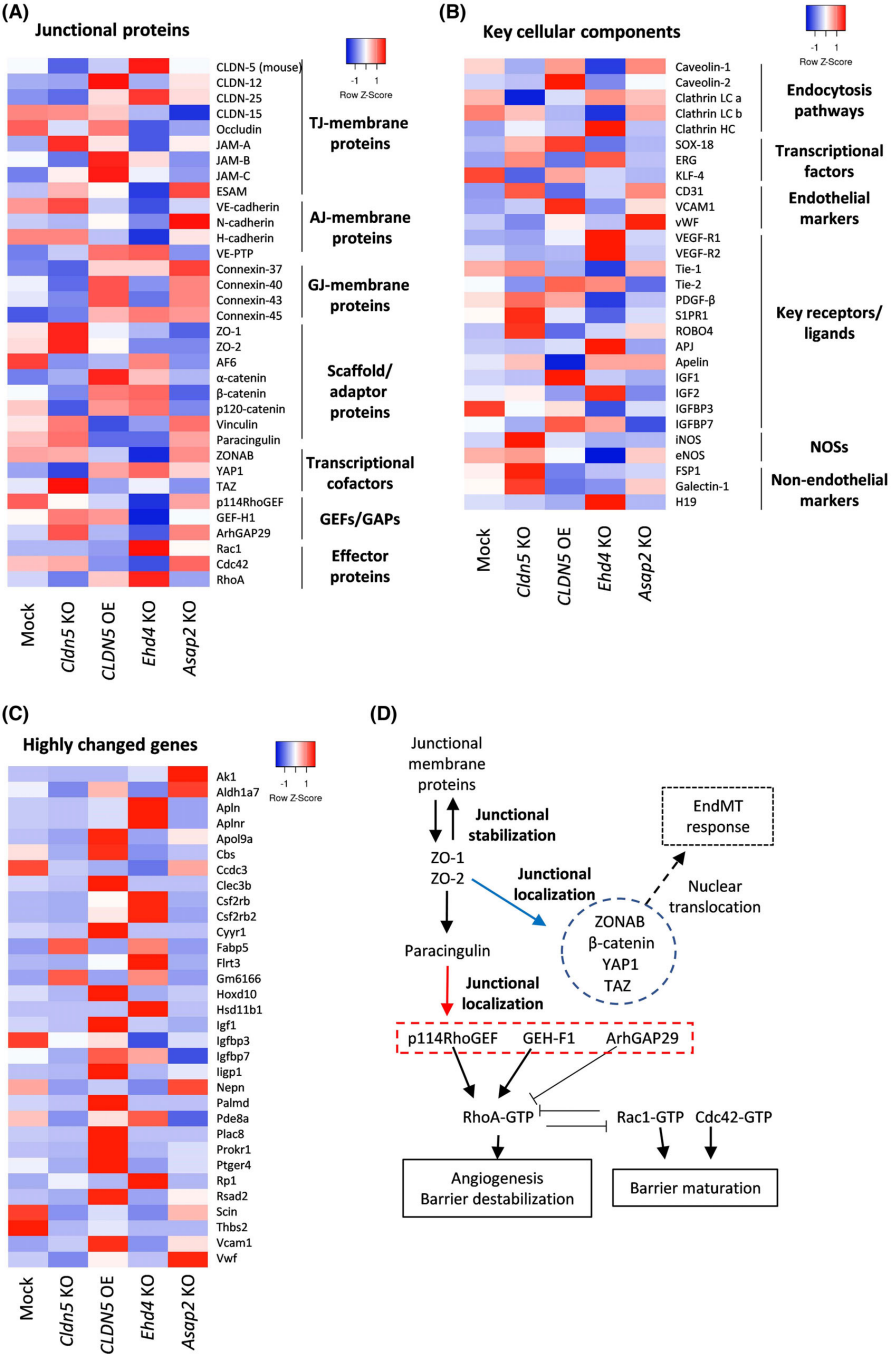
mRNA changes in individual gene knockout cells. (A–C) Heat maps of gene expression levels in the series of bEnd.3 cells. Each heat map shows the expression profiles of (A) junction‐related genes, (B) key cellular components and (C) genes that highly changed in *EHD4* or *ASAP2* KO cells. (D) Schematic illustration of junctional stabilisation‐mediated gene/protein regulation. Junctions are stabilised by the interaction/localisation of junctional membrane proteins and junctional scaffold proteins. Stabilised junctions can prevent intranuclear transportation of some transcription factors, which cause dedifferentiation by sequestering them in the junctions, and can recruit some small GTPases and their effectors to maintain the morphology of peri‐junctional actin cytoskeleton. AJ, adherens junction; EndMT, endothelial to mesenchymal transition; GAP, GTPase activating protein; GEF, guanine nucleotide exchange factor; GJ, gap junction; KO, knockout; NOS, nitric oxide synthase; OE, overexpression; TJ, tight junction.

To evaluate the mechanism by which *EHD4* and *ASAP2* inhibition stabilises CLDN‐5 expression, whole transcriptomic analysis of these cells was performed. CLDN‐5 KO and CLDN‐5 OE cells were also analysed to distinguish genes highly influenced by CLDN‐5 expression (for all data, see Table S2). CLDN‐5 expression levels highly influenced the expression of gap junction proteins, specifically connexin‐37 and ‐40, and junctional adhesion molecule (JAM)‐B (Table 1). Of note, an EndMT (endothelial to mesenchymal transition) marker, *S100A4* (encoding fibroblast‐specific protein 1), was upregulated by the loss of CLDN‐5 expression. Conversely, it was downregulated in CLDN‐5 OE cells, *EHD4* KO and *ASAP2* KO cells (Fig. 3A).

**Table 1:** mRNA levels of genes highly affected by CLDN‐5 expression in bEnd.3 cells. Genes showing two‐fold changes in both CLDN‐5 KO vs mock and CLDN‐5 OE vs mock are extracted from Table S2. Values highlighted by bold indicates that their changes may be induced by the increased cell‐surface CLDN‐5 expression rather than by the knockout of *EHD4* or *ASAP2*. EndMT, endothelial‐to‐mesenchymal transition; S1P, sphingosine‐1‐phosphate; TEM, tumour endothelial marker.

Gene	Notes	Mock	CLDN‐5 KO	CLDN‐5 OE	*EHD4* KO	*ASAP2* KO
Highly correlated with CLDN‐5 expression level						
*ACER2*	Upregulate S1P production	21.02	10.09^a^	**71.80** ^a^	17.59	28.32
*CXCL12*		214.66	72.28^a^	**953.84** ^a^	32.94^a^	**247.61** ^a^
*FAM189A2*		17.59	4.52^a^	**40.24** ^a^	**33.00** ^a^	8.43
*GAS6*		19.96	8.94^a^	**59.98** ^a^	**55.59** ^a^	3.34^a^
*GJA4*	Connexin‐37	18.34	4.58^a^	**37.08** ^a^	8.68^a^	**32.47** ^a^
*GJA5*	Connexin‐40	135.55	26.95^a^	**331.42** ^a^	8.50^a^	**284.57** ^a^
*H2‐K1*		25.08	5.83^a^	**76.55** ^a^	10.06^a^	14.69
*HACD4*		25.61	10.71^a^	**56.66** ^a^	19.66^a^	**45.23** ^a^
*HS3ST1*		11.87	1.01^a^	**36.38** ^a^	**27.45** ^a^	4.42
*IFI27L2A*		40.98	19.35^a^	**281.63** ^a^	13.93^a^	13.75^a^
*IFIT1*		25.45	10.26^a^	**58.03** ^a^	5.11^a^	**58.83** ^a^
*IFIT3*		20.80	9.04^a^	**109.79** ^a^	15.80	**66.83** ^a^
*JAM2*	JAM‐B	92.61	35.59^a^	**192.71** ^a^	**112.75** ^a^	47.37^a^
*OASL2*		14.10	1.92^a^	**53.09** ^a^	5.32^a^	30.69^a^
*RFLNB*		198.25	85.01^a^	**550.60** ^a^	**368.40** ^a^	**265.82** ^a^
*SEMA7A*		23.76	1.29^a^	**48.55** ^a^	9.02^a^	32.42
*SLC29A1*		25.47	10.64^a^	**53.41** ^a^	**105.60** ^a^	12.26^a^
*VSIR*		19.26	2.03^a^	**38.78** ^a^	**47.61** ^a^	10.31
Highly inversely correlated with CLDN‐5 expression level						
*GPRC5B*		29.29	75.02^a^	**12.08** ^a^	23.08	**15.18** ^a^
*PTPRN*	TEM	21.00	44.09^a^	**5.14** ^a^	**6.16** ^a^	26.68
*S100A4*	EndMT marker	473.90	957.05^a^	**167.08** ^a^	**302.25** ^a^	**361.17** ^a^

^a^ *q*‐value is <0.01, compared with Mock.

**Fig. 4:**
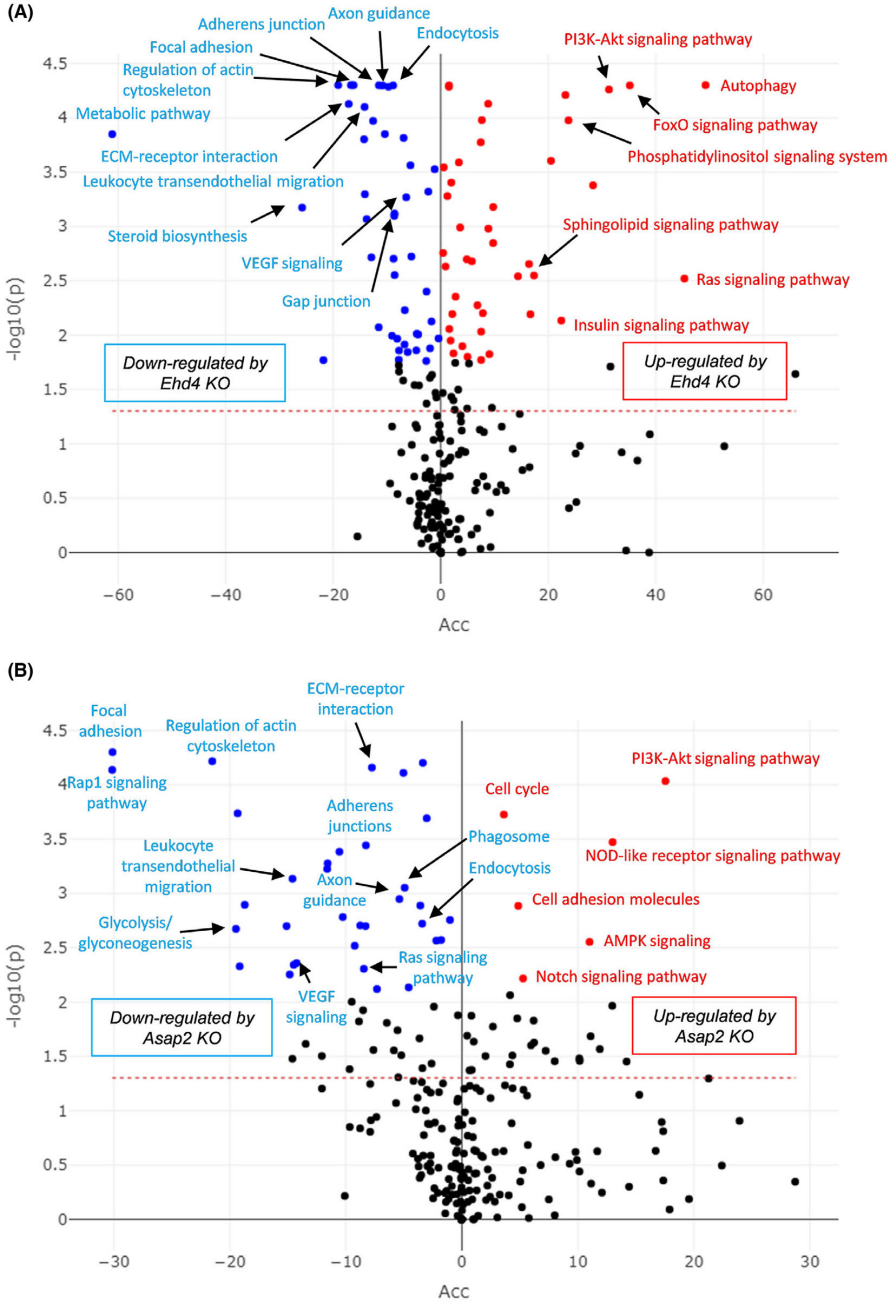
Pathways highly influenced by *EHD4* or *ASAP2* knockout. (A, B) Volcano plots to show genes highly changed by (A) *EHD4* or (B) *ASAP2* knockout. The adjusted *P*‐value (y‐axis) and the accumulation score (x‐axis) were calculated by MITHrIL (miRNA enriched pathway impact analysis) analysis (*n* = 3). All major pathways are indicated in the volcano plots.

*EHD4* and *ASAP2* inhibition regulated clearly different genes/pathways as shown by enrichment analysis (Table S2; Fig. 4A,B). The expression levels of genes that support adherens junction stability (VE‐protein tyrosine phosphatase and catenins) were upregulated in *EHD4* KO cells, while those of gap junction proteins were clearly upregulated in *ASAP2* KO cells similar to that observed in CLDN‐5 OE cells. Changes in genes related to growth factors, receptors and cellular components that induce vascular permeability are summarised in Fig. 3B,C and Table 2, and key regulators and mechanisms for EndMT responses, angiogenesis and barrier maturation are shown in Fig. 3D.

**Table 2:** Key receptors/ligands/cellular components in *EHD4* or *ASAP2* knockout cells. Key genes are extracted from Table S2. Values highlighted by bold indicates that their changes may tighten the endothelial cellular barriers.

Gene	Signalling pathways/functions	Mock	CLDN‐5 KO	CLDN‐5 OE	*EHD4* KO	*ASAP2* KO
*AK1*	AMPK signalling ↑	8.14	7.30	7.64	12.39	**46.30** ^a^
*APLN*	Apelin signalling ↑	366.63	**493.38** ^a^	74.68^a^	**537.13** ^a^	**540.27** ^a^
*APLNR*	Apelin signalling ↑	31.24	25.75	**57.41** ^a^	**319.27** ^a^	0.90^a^
*CAV1*	Caveolae‐mediated transcytosis ↑	624.15	**452.67** ^a^	708.50^a^	**286.98** ^a^	733.53^a^
*CAV2*	Caveolae‐mediated transcytosis ↑	110.02	104.75	173.00^a^	**91.95** ^a^	117.78
*CLU1*	MMP9 inhibitor	56.58	**111.12** ^a^	50.94	8.86^a^	**168.87** ^a^
*ECM1*	Angiogenic responses ↑	58.32	153.27^a^	**17.70** ^a^	175.89^a^	62.02
*EHD3*	EHD3	38.68	8.051^a^	43.98	154.37^a^	35.21
*FABP5*	Transporter of DHA	48.94	602.29^a^	56.10	521.07^a^	34.89^a^
*FLT1*	VEGF receptor 1	36.14	34.80	51.25^a^	105.04^a^	44.89
*IGF1*	GLUT1 activity ↓ VEGF signalling ↑	12.98	8.34	89.75^a^	9.40	**0.86** ^a^
*IGF2*	VEGF signalling ↑	73.91	87.89^a^	**21.84** ^a^	210.83^a^	**11.62** ^a^
*IGFBP3*	IGF1 signalling ↓	878.12	406.95^a^	505.43^a^	5.39^a^	343.13^a^
*IGFBP7*	IGF1/2 signalling ↓	35.00	19.29^a^	**68.50** ^a^	**53.75** ^a^	0.92^a^
*KDR*	VEGF receptor 2	200.54	**147.47** ^a^	**154.68** ^a^	468.61^a^	**182.55** ^a^
*LGALS1*	EndMT marker	274.37	541.42^a^	**67.63** ^a^	**105.90** ^a^	345.81^a^
*SLC2A1*	GLUT1	26.01	20.07	28.10	20.04	23.32
*VWF*	Angiogenic responses ↓	19.61	3.65^a^	**37.14** ^a^	16.72	**88.00** ^a^

^a^ *q*‐value is <0.05, compared with Mock.

In *EHD4* KO cells, apelin‐apelin receptor signalling, which promotes cellular polarisation [23], was enhanced. Interestingly, apelin signalling was potently suppressed in CLDN‐5 OE cells. *EHD3* and *EHD4* may involve the localisation of vascular endothelial growth factor (VEGF) receptors [24], indicating that increased expression levels of VEGF‐R1, −R2 and *EHD3* observed in *EHD4* KO cells may compensate for the functional impairment of VEGF receptors (Table S2; Fig. 4A).

In *ASAP2* KO cells, autocrine‐mediated insulin‐like growth factor (IGF)‐mediated signalling—which promotes endothelial angiogenesis and migration [25]—was downregulated, and the expression of von Willebrand factor—whose deficiency causes increased angiogenic responses [26]—was significantly upregulated. Added to this, the expression of *Ak1*, which reversibly catalyses the 2ADP ↔ ATP + AMP reaction and activates AMP‐activated protein kinase (AMPK), was significantly upregulated only in *ASAP2* KO cells (Fig. 4B). AMPK may tighten the endothelial barrier via a largely unknown mechanism [27]. Alternatively, it may be a compensation reaction since IGF‐1 can regulate glucose uptake by upregulating activity, not expression, of the glucose transporter 1 (GLUT1) in endothelial cells [25] and AMPK can upregulate GLUT1 function [28].

**Fig. 5:**
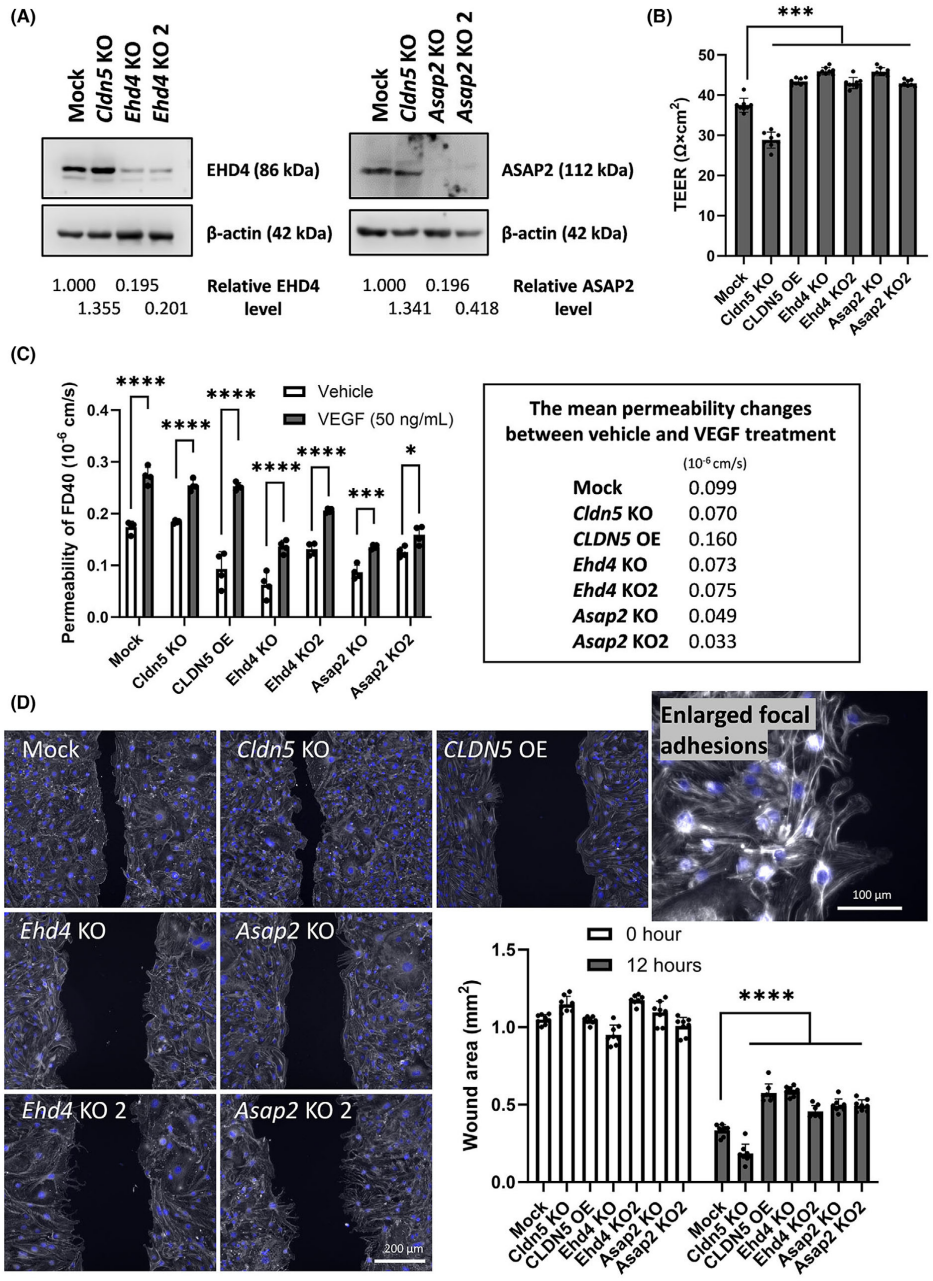
The effect of *EHD4* and *ASAP2* knockout on angiogenic responses in vitro. Another *EHD4* or *ASAP2* knockout (KO) cell line was generated by using different gRNA (guide RNA) sequences. (A) Protein expression level of generated KO cells was checked by western blotting and (B) TEER of generated cells was measured. The values below the blotting membranes are relative protein levels calculated by a densitometric analysis (*n* = 1). Values in (B) represent the mean ± SD (*n* = 8). One‐way ANOVA followed by Dunnett's multiple comparisons test: ***P < 0.0005. (C) Permeability of 40 kDa of fluorescence‐conjugated dextran (FD40) across the monolayers of these cells was assessed 24 h after the treatment of vehicle or VEGF‐A (50 ng·mL^−1^). Values represent the mean ± SD (*n* = 4) in left bar graph and the mean changes in FD40 permeability was calculated by subtraction (shown in right panel). Two‐way ANOVA followed by Šídák's multiple comparisons test: *P < 0.05; ***P < 0.0005; ****P < 0.0001. (D) Cell migration was assessed by wound‐healing assay in a series of knockout cells. Cells were stained with phalloidin and Hoechst33342. Images were taken at 0 or 12 h after the wound area was analysed and representative images at 12 h after the scratch are shown. A representative image of enlarged focal adhesion was acquired with higher magnification and is shown in top right panel. The scale bar indicates 100 (enlarged) or 200 μm. The quantified values of wound area represent the mean ± SD (*n* = 8). Two‐way ANOVA followed by Tukey's multiple comparisons test: ****P < 0.0001.

To confirm the effect of knockout of *EHD4* and *ASAP2*, additional bEnd.3 KO cells were prepared by transducing different gRNA sequences (*EHD4* KO2 and *ASAP2* KO2) (Fig. 5A) and their TEER was higher than that of mock (Fig. 5B). Although knockout of *EHD4* and *ASAP2* changed the expression of very different genes, both of them could downregulate genes central to VEGF signalling (Fig. 4A,B). As this was a central signalling mechanism identified, to check the sensitivity of VEGF to these cells, FD40 (40 kDa tracer) permeability was assessed in the presence of VEGF‐A (Fig. 5C). FD40 permeability was increased by VEGF in all cells, but this was potently attenuated in *ASAP2* KO cells. Importantly, increased CLDN‐5 protein level only was not enough to acquire the resistance against VEGF because both CLDN‐5 KO and CLDN‐5 OE cells showed similar FD40 permeability in the presence of VEGF. *EHD4* KO cells might respond to VEGF more potently compared to *ASAP2* KO cells, as *KDR* gene (VEGF‐R2) expression was likely increased in *EHD4* KO cells by a compensation mechanism (Table 2; Table S2). To further address the angiogenic response in these cells, wound‐healing assays was carried out (Fig. 5D). The migration of bEnd.3 cells was promoted by the absence of CLDN‐5 and inhibited by the overexpression of CLDN‐5. The scratch wounds in a monolayer of *EHD4* or *ASAP2* KO cells were significantly reduced compared to that of mock cells, and enlarged focal adhesions could be observed, likely due to the impairment in disassembly of focal adhesion [29, 30]. These results suggest that the inhibition of *EHD4* or *ASAP2* tighten the endothelial barrier by reducing the sensitivity to VEGF and cellular migration ability.

## Discussion

Here, we applied a genome‐wide phenotypic CRISPR screen using the GeCKO v2 library to identify specific regulators of cell‐surface CLDN‐5 expression level. Using this approach, we have identified two novel CLDN‐5 regulating genes: *EHD4* and *ASAP2*. While *EHD4* and *ASAP2* do not show EC‐specific gene expression, their expression in ECs is relatively high in both the retina and brain [1, 31]. Interestingly, both *EHD4* and *ASAP2* expression levels in mouse brain ECs are significantly lower than those in mouse lung, kidney and intestinal ECs [1, 32, 33], implying that pericytes, astrocytes or other associated cells may suppress these genes in order to tighten the endothelial barrier. *TLL1*, which encodes Tolloid‐like 1 (TLL1), was the most detected gRNA sequences in our screen. The inhibition of *TLL1* could inhibit TGF‐β signalling, the target of RepSox [6, 21], by preventing its release from the precursor complex [34]. However, *TLL1* KO cells failed to establish a tighter cellular barrier although they showed higher CLDN‐5 expression levels, probably due to the decline in other substrates of TLL1. As demonstrated by Roudnicky et al, upregulation of CLDN‐5 expression levels by inhibiting several genes does not always tighten the cellular barrier [21]. Similarly, lentivirus‐mediated overexpression of CLDN‐5 in human umbilical vein endothelial cells (HUVECs) failed to form an enhanced barrier [35], indicating that preserving other key proteins for junctional maturation is required for CLDN‐5 enhancers to stabilise the endothelial barrier.

**Fig. 6:**
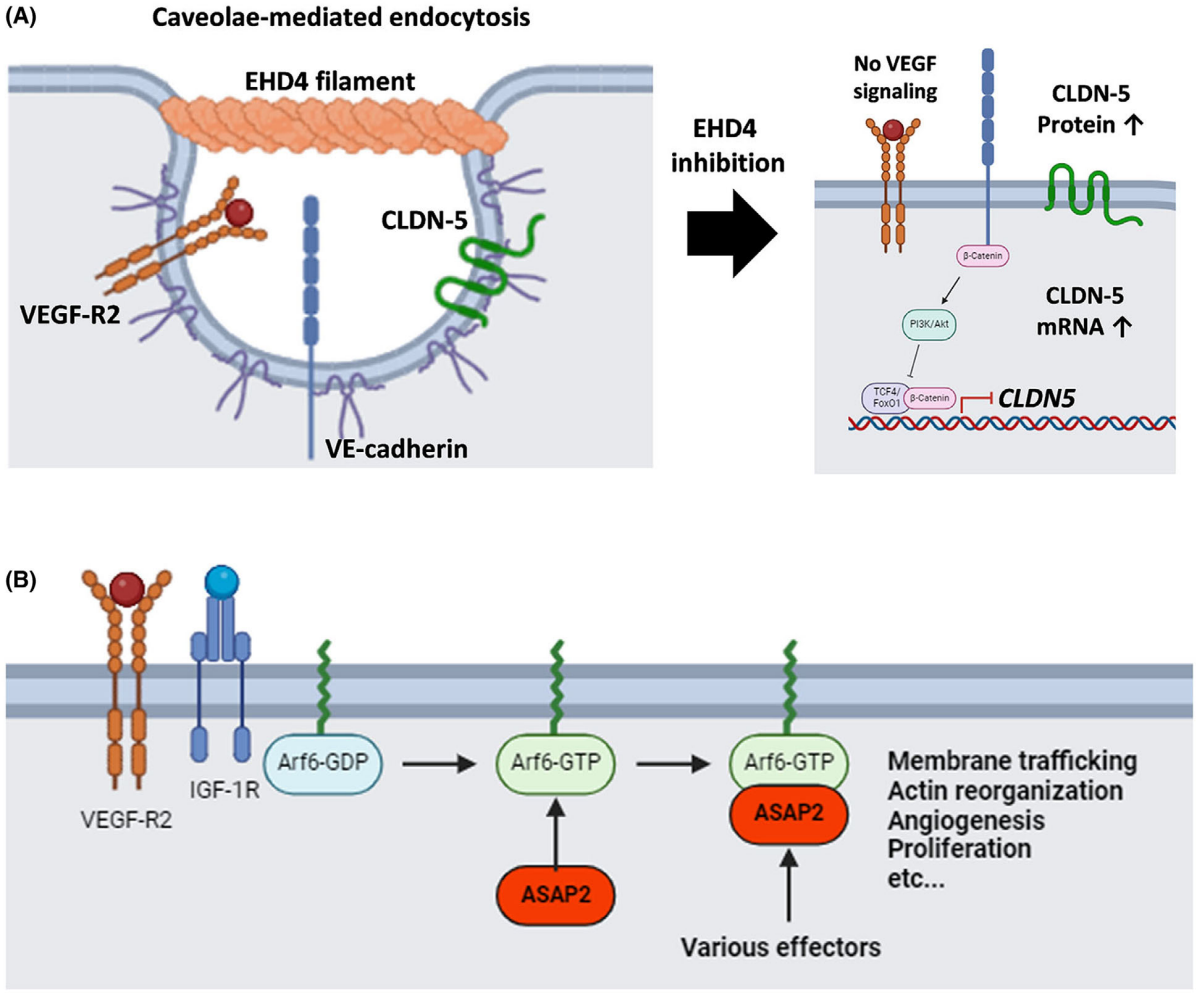
Putative mechanism of action of *EHD4* and *ASAP2*. (A, B) Schematic representation of potential mechanism of action of *EHD4* and *ASAP2*. (A) *EHD4* is known to regulate caveolae that is important for the degradation of VE‐cadherin and the downstream signalling of vascular endothelial growth factor (VEGF). The inhibition of *EHD4* may prevent the caveolae‐mediated phenomena and upregulate the transcriptional activity of the CLDN‐5 promoter. (B) *ASAP2* is known to bind to Arf6‐GTP without GTP hydrolysis. Arf6‐GDP is converted to Arf6‐GTP upon VEGF and IGF (insulin‐like growth factor) signalling. The inhibition of *ASAP2* may prevent the Arf6‐GTP‐mediated vascular destabilisation.

EHD (EH Domain‐Containing) family members 1, 2 and 4 are known to stabilise caveolae [36], which is a major endocytosis pathway of VE‐cadherin and CLDN‐5 [37, 38]; their oligomers assemble at the neck of caveolae or membrane vesicles in a ring‐shape filament (Fig. 6A) [39]. *EHD4* also associates with the trafficking of VEGF‐R2 and its deletion causes impaired angiogenic responses if the compensation by other EHD family members is not enough [24]. Knockdown of *EHD4* has been shown to suppress the migration and angiogenic response of HUVECs by preventing the removal of trans‐interacted VE‐cadherin from the adherens junctions in migrating ECs [40]. Therefore, it is highly possible that VE‐cadherin‐mediated adherens junctions in the resting *EHD4* KO cells was stabilised, and resulted in the inhibition of formation of a transcriptional repressor complex for CLDN‐5, composed of β‐catenin and FoxO1 [41]. Added to this, *EHD4* ^−/−^ mice are viable and develop retinal vasculature with equal numbers of vascular branch points, sprouts, and endothelial cell numbers, but show attenuated angiogenic responses in the early vascular development phase [40], indicating that inhibition of the *EHD4* gene can be tolerable to stabilise the neural vasculature.

The function of *ASAP2* (ArfGAP with SH3 domain, ankyrin repeat and PH domain 2) in ECs is still largely unknown. *ASAP2* expression can promote proliferation and migration of cancer cells via epithelial‐mesenchymal transition [42, 43]. As an actin regulator, ASAPs are likely involved in actin‐based endocytosis processes, focal adhesion, and stress fibre formation by direct binding to actin filaments [44]. Interestingly, ASAPs can function as effectors for Arf6 upon its activation by IGF or VEGF stimulation (Fig. 6B) [45, 46, 47, 48]. Since IGF‐1/IGF‐1R signalling forms an autocrine loop [49], there was significantly less *IGF1* and *IGF2* expression levels observed in our *ASAP2* KO cells (Table 2), potentially due to a disturbance in this loop. ASAPs can directly bind to paxillin, whose knockdown impaired focal adhesion disassembly and prevented cell migration [30, 45]. The differences in function and/or binding partners of *ASAP1* and *ASAP2* are still unclear, but they have clear differences in the binding affinity to actins [44].

Currently, known CLDN‐5 enhancers inhibit TGF‐β signalling, or activate cAMP or β‐catenin signalling [14, 15, 21, 50, 51]; all of these have no EC‐specificity. Stabilised VE‐cadherin‐mediated adherens junctions can inhibit GSK‐3β in a phosphatidylinositol 3‐kinase (PI3K)/Akt dependent manner and GSK‐3β inhibition stabilises the adherens junction by an increase in cytosolic β‐catenin levels [15], indicating that GSK‐3β inhibition can make a positive feedback loop to tighten the barrier. Therefore, both *EHD4* and *ASAP2* inhibition can also inhibit GSK‐3β and the pathway analysis clearly supported that this inhibition could enhance PI3K/Akt signalling (Fig. 4), but neither *EHD4* nor *ASAP2* are associated with TGF‐β signalling or cAMP‐mediated signalling.

This study represents the first genome‐wide phenotypic screen using a CLDN‐binding molecule to identify CLDN‐regulating genes from a knockout cell library. Theoretically, this screen has more power to identify higher numbers of CLDN‐5‐regulating genes compared to other methods. It should, however, be noted that CLDN‐5 upregulation or junctional stabilisation may attenuate cellular proliferation by contact inhibition and the majority of pooled cells that have low or normal CLDN‐5 expression levels may have proliferated well, resulting in lower screening efficacy and reproducibility of CLDN‐5^high^ cells. In general, cells need to be cultured in the confluent state for more than five days to efficiently express CLDN‐5 on their cell‐surface, but it is highly possible that CLDN‐5^high^ cells would be pushed away by highly proliferating cells during this process. This is a limitation of random gene knockouts from whole genes. Another limitation is a lack of proper, non‐heterogenic, immortalised neural microvascular ECs. ECs with much stronger TJs would be more sensitive to the detection of CLDN‐5 regulating genes by a phenotypic screen. However, many in vitro brain and retinal Ecs, including bEnd.3 cells, do not develop strong TJs like those observed in vivo. It could, however, be argued that starting with cells displaying a low barrier phenotype and then driving a stronger barrier phenotype will identify the more profound barrier‐regulating genes. In effect, this is what we sought to examine.

Here, we performed a genome‐wide phenotypic screen to identify genes that specifically and negatively regulate cell‐surface expression levels of CLDN‐5. The two discovered genes, *EHD4* and *ASAP2*, were clearly novel CLDN‐5 regulators. Of note, *ASAP2* would not have been identified using a transcriptional reporter‐based screening because *ASAP2* inhibition did not change CLDN‐5 mRNA levels. There are still no reports about missense mutations into the *EHD4* or *ASAP2* genes, but these mutations may counteract vascular destabilisation. Added to this, at present, only RNA interference or CRIPR/Cas9‐based gene inhibition are modifiers of *EHD4* or *ASAP2* expression/function, since there are no chemical inhibitors of *EHD4* or *ASAP2* except for niclosamide, which downregulated *ASAP2* expression in pancreatic cancer cells [42]. However, in our hands, niclosamide could not regulate *ASAP2* expression or tighten the cellular barrier in bEnd.3 cells (data not shown). From these findings, screens of chemical inhibitors of *EHD4* and *ASAP2* will be carried out to develop CLDN‐5 upregulation‐based vascular stabilisers. Importantly, inhibitors of these components will not need to bypass the BBB as the target tissue will be the microvascular ECs of the brain or retina. Therefore, systemic or local delivery in the form of small molecules, antibodies or oligonucleotides targeting *EHD4* and/or *ASAP2* may represent highly novel approaches to treating neurological and ophthalmological conditions in the future.

## Materials and methods

### Cells

Mouse immortalised brain endothelial cell line (bEnd.3 cells; RRID: CVCL_0170) (CRL‐2299, American Type Culture Collection) was maintained in Dulbecco's modified Eagle's medium (DMEM) supplemented with 10% fetal bovine serum (FBS) (Sigma‐Aldrich, Saint Louis, MO, USA), 100 U·mL^−1^ penicillin, and 100 μg·mL^−1^ streptomycin (Sigma‐Aldrich). All cells were incubated at 37 °C under 5% CO_2_ and all experiments were performed with mycoplasma‐free cells. bEnd.3 cells were infected with LentiArray Cas9 lentivirus (Thermo Fisher Scientific, Waltham, MA, USA), and single colony‐derived bEnd.3 cells expressing CRISPR/Cas9 (bEnd.3/Cas9) were prepared by a limiting dilution method. Then, 1.0 × 10^6^ bEnd.3/Cas9 cells were seeded into T75 flask (Sarstedt, Nümbrecht, Germany) a day before infection of mouse GeCKO2 (Genome‐Scale CRISPR Knock‐Out) lentiviral pools (Sigma‐Aldrich). The 100 × 10^6^ and 30 × 10^6^ transduction units of lentiviral pool library A and B were treated at 80% cell confluency, respectively. Two days after the infection, cells were treated with 5 μg·mL^−1^ puromycin (InvivoGen, San Diego, CA, USA) to remove non‐infected cells, and 50% of cells were dead by puromycin. To prepare a cell monolayer of pool bEnd.3 cells, 12 × 10^5^ cells were seeded onto collagen type I (Sigma‐Aldrich)‐coated 6‐well cell‐culture inserts (TC inserts, 0.4 μm pore size, polyester membrane; Sarstedt) and incubated for at least seven days.

### Plasmid vectors and transfection

A pU6 vector (Millipore‐Merck, Darmstadt, Germany) with puromycin‐resistance gene under the CMV promoter was used to construct a guide RNA (gRNA)‐expressing vector using BamHI/BsrGI site. All primer sequences for constructing these vectors are summarised in Table S3. pcDNA3.1 expressing human CLDN‐5 was prepared previously [13]. The vectors were transfected into cells using FuGENE HP DNA (Promega, Madison, WA, USA) and cells were cultured for more than 10 days with 10 μg·mL^−1^ Blasticidin S (InvivoGen) or 5 μg·mL^−1^ puromycin. Several transfectants were cloned by a limiting dilution method.

A C‐CPEmt‐expressing vector was used to prepare recombinant His‐tagged C‐CPEmt according to the method as described previously [20]. The apparent purity of C‐CPEmt was examined by SDS‐PAGE, followed by Coomassie brilliant blue gel staining.

### FACS‐based phenotypic screen

The KO cells cultured onto TC inserts (0.4 μm pore size, polyester membrane; Sarstedt) were harvested by mild trypsin treatment and aggregated cells were removed by passing through a cell strainer (100 μm mesh). Then, 1.0 × 10^6^ cells were incubated with the mixture of 20 μg C‐CPEmt and 2 μg anti‐His tag antibody (clone 9C11, Wako Pure Chemical, Osaka, Japan) in 1% BSA‐PBS for 1 h at 4 °C. Cells were washed in PBS once by centrifugation and then incubated with Alexa488‐conjugated anti‐mouse antibodies (Thermo Fisher Scientific) for 1 h at 4 °C. Cells were washed twice in PBS by centrifugation and resuspended in 0.2% BSA‐PBS with 2 mm EDTA. A population of 1% of the total cells with the highest fluorescence intensity was isolated by FACSAria Fusion (BD Biosciences, Franklin Lakes, NJ, USA). Isolated cells were cultured for seven days and their genomic DNAs were then isolated by GeneJet Genomic DNA purification kit (Thermo Fisher Scientific). The gRNA sequences inserted into genome DNAs were amplified by PCR using primers listed in Table S3. Sequencing of the PCR products was carried out using Illumina NovaSeq 6000 platform (2 × 150 bp) at Eurofins GATC Biotech (Konstanz, Germany). The detected sequences were counted by QIAGEN CLC genomics workbench (Qiagen, Hilden, Germany) and the gRNA sequence library of GeCKO2 was used for a reference. One mismatch was tolerated if a non‐mismatched sequence was also detected.

To check the cell‐surface expression level of CLDN‐5 in single gene KO bEnd.3 cells, Accuri C6 flow cytometer (BD Biosciences) was used. Cells were maintained in a T25 flask at a confluent state for seven days before flowcytometric analysis to maximise C‐CPEmt binding. The mean fluorescence intensity (MFI) was evaluated by C6 software or flowing software [52] and cells with 1.2‐fold higher MFI compared to mock cells were selected as the candidate genes.

### Measurement of the integrity of paracellular barriers

The measurement of the integrity of paracellular barriers was performed as previously described [13]. To prepare a cell monolayer of bEnd.3 cells, 0.8 × 10^5^ cells were seeded onto collagen type I coated 24‐well TC inserts and incubated for at least seven days. Some blank inserts (without cells) coated with collagen type I were also cultured. The medium was exchanged every two days. To check the anti‐angiogenic response of the bEnd.3 monolayers, 50 ng·mL^−1^ of human VEGF‐165 (PeproTach, Thermo Fisher Scientific) was treated in both chambers 24 h before the measurement. Trans‐endothelial electrical resistance (TEER) was measured by using a Millicell ERS Ohmmeter (Millipore, Bedford, MA, USA). The cells were incubated for 15 min at room temperature, and then electrical resistance (Ω_sample_ and Ω_blank_) was measured. TEER was calculated by using the following equation;

$$\text{TEER}\left(\Omega \times {\mathrm{cm}}^{2}\right)=\left(\Omega_{\text{sample}}-\Omega_{\text{blank}}\right)\times \text{culture area of}\;\mathrm{TC}\;\text{inserts}\left(0.3\;{\mathrm{cm}}^{2}\right)$$

To measure the permeability of fluorescein isothiocyanate–labeled dextran with an average molecular weight of 4 kDa (FD4; Sigma‐Aldrich) or 40 kDa (FD40; Sigma‐Aldrich) across the monolayers, cell culture inserts were washed by DMEM (without FBS) and transferred to 24‐well plates (Sarstedt) containing 1.0 mL of DMEM (without FBS). The DMEM in the top chamber was then changed to 1 mg·mL^−1^ FD4 or FD40 in DMEM (without FBS), and the culture inserts were incubated for 60 min at 37 °C. Samples were collected from the bottom compartment, and the concentration of the tracer was measured using FLUOstar OPTIMA plate reader (BMG Labtech, Ortenberg, Germany). Apparent permeability coefficients were calculated by using the following equation:

$$P_{\mathrm{app}}\left(\mathrm{cm}/s\right)=\begin{array}{l} (\text{volume of bottom chamber}\\ \times \text{tracer concentration in bottom chamber})\\ /(\text{culture area}\times \text{initial tracer concentration}\\ \times \text{incubation time}) \end{array}$$

### General assessment of individual gene KO cells

Cells were cultured onto 6‐well plates or chamber slides with type I collagen coating for seven days at a confluent state. The following assays were then carried out as previously described [6, 13, 53], with a few minor modifications. For RNA‐sequencing or real‐time RT‐PCR, cells were lysed in lysis buffer for RNA isolation. For immunocytochemistry, cells were fixed in 4% PFA for 15 min and then permeabilised in 0.1% Triton‐X for 5 min. After washing, blocking was carried out using 1% BSA‐PBS followed by incubation with primary antibodies (4C3C2 mouse anti‐CLDN‐5 antibody (Thermo Fisher Scientific) and rabbit anti‐ZO‐1 polyclonal antibody (40–2200, Thermo Fisher Scientific) in 1% BSA‐PBS. Cells were then washed twice with PBS and incubated with Alexa488‐conjugated goat anti‐mouse IgG secondary antibody and Cy3‐conjugated goat anti‐rabbit IgG secondary antibody (Thermo Fisher Scientific) for 2 h at room temperature and counterstained with Hoechst33342 to visualise nuclei. Images were acquired on a Zeiss LSM 710 confocal microscope (Carl Zeiss, Oberkochen, Germany) with EC Plan‐Neofluor 20×/NA 0.50 M27 or Plan‐Apochromat 63×/NA 1.40 Oil DIC M27 objective lens. The number of cells with discontinuous ZO‐1 localisation or intensive ‘zig‐zag’ ZO‐1 staining at the cell–cell border was quantified by manual counts. Five images were used for quantification.

For protein fractionations by Triton‐X solubility, cells were lysed in 1% Triton‐X in PBS with phosphatase and protease inhibitors (Millipore‐Sigma, Burlington, MA, USA), incubated for 30 min on ice and centrifuged at 12 000 ***g*** at 4 °C for 20 min to collect the supernatant (Triton‐X soluble fraction). The pellet was washed in 1% Triton‐X in PBS with centrifugation and resuspended in 20 μL of 1% Triton‐X in PBS. Insoluble pellet was then lysed in 4X SDS‐PAGE loading buffer (final SDS concentration 2%) with boiling at 95 °C for 10 min (Triton‐X insoluble fraction).

For whole protein isolation, RIPA lysis buffer with phosphatase and protease inhibitors was used to prepare the cell lysates.

For scratched wound assay, the confluent bEnd.3 cells or primary mouse brain ECs on 6‐well‐plates were wounded using a 200 μL pipette tip to create a cell‐free zone and cells were washed with PBS twice and cultured for 12 h. Subsequently, cells were fixed in 4% PFA, permeabilised with 0.1% Triton‐X100, and then stained with Alexa488 phalloidin (Thermo Fisher Scientific) and Hoechst33342. The images were acquired using Revolution (ECHO) with 4× Plan Fluorite/NA 0.13 or 20× Plan Achromat/NA 0.40 objective lens for quantification of wound areas by the plug‐in ‘wound healing size tool’ in ImageJ.

### Western blotting

Protein concentrations in lysates were determined by using the BCA assay kit (Thermo Fisher Scientific). Protein lysate (20 or 50 μg) were separated by SDS polyacrylamide gels. Proteins were transferred to methanol‐activated polyvinylidene difluoride membranes (Immobilon‐P, Millipore‐Merck) and blocked for 1 h at room temperature in 5% non‐fat milk and Tris‐buffered saline containing 0.05% Tween‐20 solution (T‐TBS). Membranes were incubated with primary antibodies listed in Table S3 overnight at 4 °C; washed twice for 5 min each in T‐TBS; and incubated with HRP‐conjugated goat secondary antibodies for 2 h at room temperature. After four 5 min washes in T‐TBS, protein bands were visualised using enhanced chemiluminescence (Advansta, San Jose, CA, USA) with an image analyser (C‐DiGit scanner, LI‐COR, Lincoln, NE, USA). Densitometry was performed using ImageJ with protein bands of interest normalised to the loading control β‐actin.

### Real‐time RT‐PCR and RNA‐sequencing

RNA was isolated from cells using an EZNA total RNA kit (Omega Bio‐tek, Norcoss, GA, USA) and residual DNA was completely digested by DNase I on column. cDNA was prepared using High Capacity cDNA Reverse Transcription Kit (Applied Biosystems, Foster City, CA, USA). Real‐time RT‐PCR was carried out using a FastStart Universal SYBR Green Master (ROX) master mix (Roche, Basel, Switzerland) according to the manufacturer's instructions. PCR was performed in a Step‐One Plus Real‐Time PCR instrument (Applied Biosystems). RT‐PCR conditions were as follows: 95 °C for 10 min, 37 cycles of 95 °C for 15 s, and 60 °C for 30 s. A melt curve stage was added: 95 °C for 15 s, 60 °C for 1 min, and 60–95 °C for 15 s. The comparative ΔΔCt method was used to quantify changes in mRNA levels between treatment groups. All primer sequences for real‐time RT‐PCR are summarised in Table S3.

RNA sequencing and data processing were performed at Macrogen Europe using TruSeq Stranded mRNA LT Sample Prep Kit. Gene counts were normalised using fragments per kilobase of transcript per million mapped reads (FPKM) and genes with more than 1 FPKM were considered as detected genes. The two‐stage step‐up method of Benjamini, Krieger, and Yekutieli was applied by GraphPad Prism 10 to compare two groups with each other. Values of the corrected *P*‐value (*q*‐value) lower than 0.01 were considered statistically significant. Subsequently, the data were uploaded to the freely available web server heatmapper.ca [54]. The DESeq2‐based differential expression analysis and pathway analysis were performed using RNAdetector [55].

### General statistical analyses

GraphPad Prism 10 (GraphPad Software) was used for statistical analyses. Statistical analysis was performed using Student's *t*‐test, with significance represented by a *P* value of ≤0.05. For multiple comparisons, ANOVA was used with a Dunnett, Šídák or Tukey post‐test, and significance was represented by a *P*‐value of ≤0.05.

## Conflict of interest

P. Westenskow is an employee of Roche. Trinity College Dublin owns a patent portfolio related to regulation of the BBB/BRB to treat disease.

## Author contributions

YH: Designed experiments, performed experiments and wrote the original draft. GP: Performed RNA‐sequencing. NH: Performed RNA‐sequencing. JO: Developed and modified software for analysis. NH: Performed imaging analysis. CD: Performed RNA‐sequencing. MD: Supervised experiments and reviewed/edited the manuscript. PW: Supervised experiments and reviewed/edited the manuscript. MC: Supervised experiments, and reviewed/edited the manuscript.

## Supporting information


**Table S1.** Genome‐wide phenotypic screening.

**Table S2.** RNA‐sequencing of the series of bEnd.3 cells.

**Table S3.** List of reagents.

## Data Availability

The data that support the findings of this study are available from the corresponding author [campbem2@tcd.ie] upon reasonable request.
